# Advances in immunomodulation for organ transplantation: The role of the Annexin A1/FPR axis

**DOI:** 10.1016/j.clinsp.2025.100626

**Published:** 2025-03-26

**Authors:** Rafael André da Silva, João Vitor Ferreira de Lima, Raquel Fink Lins e Silva de Macedo, Monielle Sant'Ana, Cristiane Damas Gil, M. Natalia Vergara

**Affiliations:** a Biosciences Graduate Program, Instituto de Biociências, Letras e Ciências Exatas, Universidade Estadual Paulista (UNESP), São José do Rio Preto, SP, Brazil; b CellSight Ocular Stem Cell and Regeneration Research Program, Sue Anschutz-Rodgers Eye Center, University of Colorado School of Medicine, Aurora, CO, USA; c Centro Universitário Maurício de Nassau (Uninassau) ‒ Curso de Medicina, Unidade Boa Viagem, Recife, PE, Brazil; d Department of Clinical Medicine, Faculdade de Ciências Médicas da Santa Casa de São Paulo, São Paulo, SP, Brazil; e Nacional Laboratory of Bioscience ‒ LNBio, Brazilian Center for Research in Energy and Materials (CNPEM), Campinas, SP, Brazil; f Department of Morphology and Genetics, Escola Paulista de Medicina, Universidade Federal de São Paulo (UNIFESP), São Paulo, SP, Brazil

The main obstacles in organ transplants are immunological. Patients undergoing transplantation usually have local or systemic inflammation, and the procedure can exacerbate this condition, increasing the risk of rejection.^1^ The use of immunosuppressants is of great importance to the success of transplants; however, it can lead to nephrotoxicity, infections, neoplasms, and infertility.^2^

The search for new immunosuppressive agents with fewer side effects is fundamental to improving clinical outcomes in transplantation, ensuring greater efficacy and safety for patients. Annexin A1 (ANXA1) is a protein expressed in various cells and tissues and plays a crucial role in regulating inflammation and tissue repair.^3^ This protein is induced by glucocorticoids and acts by inhibiting cytosolic Phospholipase A2 (cPLA2), reducing the production of inflammatory eicosanoids and the formation of reactive oxygen species.^4^ In addition, ANXA1 promotes neutrophil apoptosis and facilitates their phagocytosis by macrophages, contributing to the resolution of inflammation and tissue homeostasis.^5^

The activity of ANXA1 is mediated by the Formyl Peptide Receptor (FPR), which plays an essential role in regulating inflammation and the immune response.^6^ The activity of FPRs is ligand-dependent, with. FPR2 exhibits the capacity to drive either pro-inflammatory or anti-inflammatory pathways based on ligand-specific conformational changes that lead to distinct downstream signaling effects.^7^^,^^8^ Given its dual role in modulating inflammation and promoting angiogenesis, the ANXA1/FPR axis has gained attention as a promising therapeutic target for enhancing transplant outcomes and reducing immune-mediated graft rejection.^3^^,^^6^

In kidney transplantation, ischemia-reperfusion injury is a major concern, exacerbating inflammation and contributing to graft dysfunction. ANXA1, through its interaction with FPRs, has shown a protective effect against acute nephrotoxicity caused by the immunosuppressants Cyclosporine and tacrolimus, reducing the migration of macrophages into the kidney tissue.^9^^,^^10^ Treatment with the AnxA1 protein mimetic peptide, Ac_2–26_, reduces macrophage infiltration in the renal ischemia-reperfusion model.^11^

Notably, FPR blockade is also associated with the induction of immune tolerance, a crucial factor in minimizing transplant rejection. In a model of acute kidney transplant rejection, FPR1 inhibition reduced neutrophil infiltration into the graft and the content of Neutrophil Extracellular Traps (NETs).^12^ In a renal ischemia-reperfusion model, FPR2 blockade reduces cell death, neutrophil migration to renal tissue, and blood circulation, resulting in a decrease in pro-inflammatory cytokines such as IL-1β, IL-8, IL-6, and TNF-α. Additionally, it negatively modulates the Mitogen-Activated Protein Kinase (MAPK) pathway.^13^ Thus, modulation of the ANXA1/FPR axis may represent a promising therapeutic strategy for reducing the incidence of rejection and improving the clinical results of kidney transplant patients.

Pancreatic islet and pancreas transplantation, often performed in conjunction with kidney transplantation for diabetic patients, also benefits from ANXA1/FPR axis modulation.^14^ Pancreatic islet transplantation has some limitations, including the need for multiple donations to a single recipient, cell loss after infusion, and the requirement for continuous immunosuppression to avoid rejection.^14^ Mesenchymal Stem Cells (MSCs), which secrete ANXA1, can improve the survival and functionality of transplanted pancreatic islets and pancreas by exerting immunomodulatory and pro-angiogenic effects, reducing the potential for rejection. *In vitro*, treatment of human pancreatic islets before transplantation with an MSC-derived cocktail composed of recombinant ANXA1, stromal cell-derived factor-1, and complement component C3a reduces apoptosis and increases insulin control.^15^^,^^16^ After transplanting pancreatic islets into immunodeficient mice with streptozotocin-induced diabetes, treatment with the cocktail improves the animals' glycemic control.^16^

In corneal transplantation, the most frequently performed human tissue transplant and the most successful allogeneic transplant worldwide, immune rejection remains a major challenge. Trauma, inflammation, or infection can induce corneal neovascularization, significantly increasing the risk of rejection.^17^^,^^18^ In eye diseases, ANXA1 has great therapeutic potential.^19^ In a model of corneal transplantation in mice, modulation of FPR2 through treatment with Resolvin D1 improved the clinical score of the animals, reducing inflammation, neovascularization, and lymphatic vessels, and reducing opacity compared to control animals.^20^

Skin transplantation, frequently employed in cases of extensive burns and chronic ulcers, is highly immunogenic, making rejection a significant concern.^21^ ANXA1 and its mimetic peptide Ac_2–26_ have great potential for improving the results of skin transplants. Ac_2–26_ reduces neutrophil infiltration, inhibits the production of pro-inflammatory cytokines such as TNF-α, IL-1β, and IL-6, and stimulates the production of angiogenic factors such as VEGF-A, which are essential for the formation of new blood vessels and proper revascularization of the graft. These processes contribute to the nutrition and oxygenation of the transplanted tissue, favoring its integration.^22^^,^^23^

In an experimental skin transplant model, AnxA1 demonstrated the ability to modulate exacerbated inflammation, promoting a more favorable environment for tissue repair and reducing the risk of inflammatory complications and rejection. The peptide Ac_2–26_, in addition to potentiating angiogenesis, improves the quality of the vascular network formed, which speeds up graft integration.^22^ It also stimulates the proliferation and migration of fibroblasts, which are essential for the production of extracellular matrix and the restoration of skin integrity.^22^ This synergistic interaction between inflammation regulation and angiogenesis promotion fosters a microenvironment that supports epithelial regeneration, mitigates the risk of fibrotic scar formation, and enhances the structural and functional integrity of the repaired tissue.

Lung transplantation, which presents unique challenges due to continuous exposure to environmental pathogens and mechanical stress,^24^ also benefits from targeting the ANXA1/FPR axis.^24^ After lung transplantation in mice, treatment with Cyclosporine H (Cy-H), an FPR1 inhibitor, reduces alveolar damage and neutrophilia. In addition, FPR1 knockout animals were transplanted and showed results with pharmacological inhibition of FPR1. It has been suggested that FPR1 promotes airway neutrophilia, but not neutrophil recruitment to other graft tissues.^25^ Similar findings are also evident in FPR2 knockout animals.^26^

Transplants still face numerous clinical challenges, especially related to rejection, exacerbated inflammatory response, and insufficient revascularization, which compromise graft integration and patient recovery.^1^ Traditional strategies are not always effective in controlling these problems, highlighting the need for new therapeutic approaches. Treatment with ANXA1 protein derivatives or modulation of FPRs resolves tissue inflammation, reducing inflammatory infiltrates and protecting against immunosuppressant-induced damage. In this context, ANXA1 has emerged as a promising alternative immunosuppressant, due to its ability to regulate inflammation and stimulate key processes in tissue regeneration, such as angiogenesis and cell proliferation.

## CRediT authorship contribution statement

**Rafael André da Silva:** Conceptualization, Investigation, Visualization, Writing – original draft. **João Vitor Ferreira de Lima:** Writing – original draft. **Raquel Fink Lins e Silva de Macedo:** Visualization, Writing – original draft. **Monielle Sant'Ana:** Conceptualization, Investigation, Visualization, Writing – original draft. **Cristiane Damas Gil:** Supervision, Writing – original draft, Writing – review & editing. **M. Natalia Vergara:** Supervision, Writing – original draft, Writing – review & editing.

## Declaration of competing interest

The authors declare no conflict of interest. The funders had no role in the design of the study; in the writing of the manuscript, or in the decision to publish.
